# Challenges and perspectives in research and teaching of host pathogen interaction topics: new post-pandemic times to Brazil and other South American countries

**DOI:** 10.1590/0074-02760220212

**Published:** 2023-05-22

**Authors:** Marcel I Ramírez, Rita de Cassia Ruiz, Gessilda de Alcantara Nogueira-Melo, Luiz Claudio Miletti, Mauro Cortez, Melyssa Negri, Giuseppe Palmisano, Jorge González

**Affiliations:** 1 Fundação Oswaldo Cruz-Fiocruz, Instituto Carlos Chagas, Extracellular Vesicles and Host-Parasite Interactions Research Group, Laboratório de Biologia Molecular e Sistemática de Tripanossomatideos, Curitiba, PR, Brasil; 2 Instituto Butantan, Laboratório de Bacteriologia, São Paulo, SP, Brasil; 3 Universidade Estadual de Maringá, Maringá, PR, Brasil; 4 Universidade do Estado de Santa Catarina, Laboratório de Bioquímica de Hemoparasitas e Vetores, Lages, SC, Brasil; 5 Universidade de São Paulo, Instituto de Ciências Biomédicas, Departamento de Parasitologia, São Paulo, SP, Brasil; 6 Universidad de Antofagasta, Unidad de Parasitología Molecular, Departamento de Tecnología Médica, Antofagasta, Antofagasta, Chile

**Keywords:** active learning, critical thinking, higher education, host-pathogen interaction, pandemic, remote education

## Abstract

Here is our proposal to improve learning in biomedical sciences for graduate and undergraduate courses with a broad vision integrating disciplines such as molecular cell biology, biochemistry, and biophysics around concepts of pathogen interaction within vertebrate and invertebrate hosts. Our paradigm is based on the possibility offered by the pandemic to have remote activities that give access to students and researchers from different places in Brazil and Latin American countries to discuss science. A multidisciplinary view of host-pathogen interaction allows us to understand better the mechanisms involved in the pathology of diseases, as well as to formulate broad strategies for the diagnosis, treatment, and control of thereof. The approach to integrating heterogeneous groups in science involves the critical analysis of national scientific resource distribution, where only some have the possibilities to conduct competitive scientific research. Solid theoretical training, contact, collaboration with groups of excellence, and training within a multidisciplinary network are our proposals for a permanent platform of scientific strengthening and dissemination for Latin America. Here we will review the concept of host-pathogen interaction, the type of institutions where it is taught and researched, new trends in active teaching methodologies, and the current political context in science.

In general, the development of Brazilian and Latin American science is affected by multiple aspects. One of them is isolation, due to the extensive geographical distances between the different centers and universities that develop frontier research in biological or biomedical sciences areas.

During the severe acute respiratory syndrome coronavirus 2 (SARS-CoV-2) pandemic, in an unsuspected way, we had the opportunity to feel connected, in spite of physical distance. Using platforms such as Teams, Google Meet, and Zoom, among others, allowed us to connect, meet, and participate in seminar series, discussing science like never before. With the help of these platforms, exciting ideas were born, among them, the Host-Pathogen Interaction Meeting I (HPIM I) in virtual form.^1^ The initiative, which will be performed annually, represents the conceptual framework of joining strategies to strengthen teaching and research on the interaction between pathogens and hosts, integrating science between different countries of the world, as shown in Fig. 1.

**Fig. 1:**
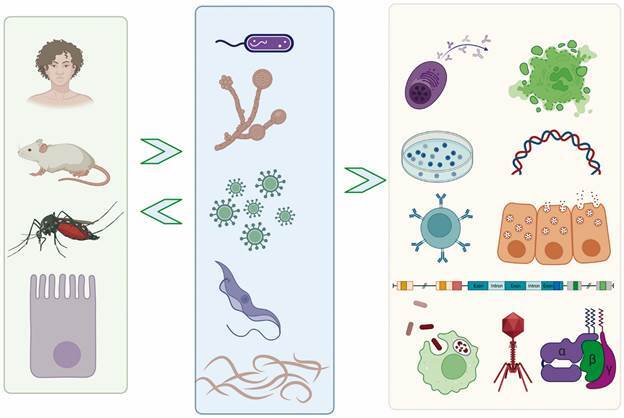
schematic representation of the interaction of pathogens (viruses, microbes, fungi and parasites) with invertebrate and vertebrate bones. A broad multidisciplinary vision of study including cell biology, biochemistry, molecular biology, immunology among other disciplines.

The host-pathogen interaction concept

Infectious diseases are caused by pathogenic microorganisms, such as viruses, bacteria, parasites, or fungi spread directly or indirectly from one person to another, which can affect people all over the world, as has been observed with the coronavirus disease 2019 (COVID-19) pandemic.^1^ ^,^ ^2^ On the other hand, neglected tropical diseases (NTDs) are a diverse group of conditions mainly prevalent in tropical areas, disproportionately affecting impoverished communities. Many of them are vector-borne, have animal reservoirs, and are associated with complex life cycles.^3^ All these factors make their control challenging. These diseases cause devastating health, social and economic consequences to more than one billion people. The gravity and result of the infection depend on the microorganism’s virulence, the host immune response and the site of infection.^3^ ^,^ ^4^ Although they are not exclusive to underdeveloped countries, they are of little financial appeal to the large pharmaceutical industry, since they do not reach the large consumer markets found in developed countries. In Brazil and Latin America, the main NTDs are: dengue, Chagas disease, leishmaniasis, malaria, schistosomiasis, leprosy and tuberculosis.^5^ ^,^ ^6^

Human pathogens are multicellular, unicellular, or acellular organisms, including bacteria, parasites, fungi, and viruses. Some of them must necessarily live in compartments of our organism or within our cells.^7^ Others may have a more transient relationship and only settle to become pathogenic where they can use animals as reservoirs or can be found in the environment.

During the interaction between host cells and pathogens, the expression of molecules involved in adhesion, invasion, proliferation, and resistance or evasion of the immune response appears to play a critical role. This set of molecules, usually referred as virulence factors, are often expressed on the cell surface of the pathogen or excreted/secreted in vesicles.^8^ In addition, the virulence and pathogenicity of a given pathogen may also be influenced or enhanced in different ways by the interaction of the pathogen with other pathogens.^9^ ^,^ ^10^ However, the concept of pathogen and virulence could be much broader and more complex than expected. Indeed, pathogenicity and virulence should be analysed as a dynamic relationship between the pathogen and its host. Thus, assigning the denomination of the pathogen to an entity that causes a specific pathology is a reductionist view that omits a much more complex reality that must necessarily consider ecological, immunological and evolutionary aspects. From this perspective, pathogenicity may be somewhat transient and depend on ecological changes and evolutionary modifications.^11^ Even more important is to understand the concept that different pathogens interacting with humans have been one of the main drivers of their evolution.^12^

Finally, assuming that a pathogen must necessarily interact with its host requires that this interaction would depend on the intrinsic characteristics of both assuming that a pathogen must necessarily interact with its host, this interaction would depend not only on the intrinsic characteristics of the pathogen but also on those of the host. Thus, each interaction may have unique and particular characteristics. This interaction varies from the eradication of the pathogen to the death of the host, passing through intermediate states in which the pathogen could become commensal without being able to cause damage to the host.^13^ The understanding of diseases derived from host-pathogen interaction is carried out in higher education institutions where undergraduate and graduate courses are fundamental to deliver concepts and strategies to improve knowledge and stimulate basic and applied research to help dissect pathogenic processes and biological characteristics of diseases.

A perspective of knowing, learning, and researching the disease that goes through a genetic, immunological, biological, biochemical, physiological, and pharmacological vision is essential. This broad perspective generates appropriate conditions to develop virtual strategies focused on strengthening the teaching and research in multiple areas in which pathogens interact with their hosts (Fig. 2).

**Fig. 2:**
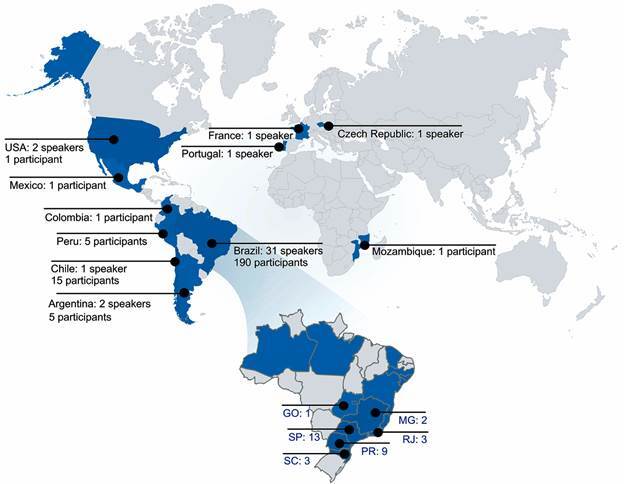
world map representing the countries of origin of participants and speakers, based on their current link with an educational and/or research institution. A highlight was made in Brazil to indicate participants (stained in blue) and speakers (marker) from different states.

Understanding Brazilian and Latin American institutions

Universities are educational institutions that belong to a concrete sociopolitical model. It is well known that the principal purpose of these institutions is to create knowledge for society and contribute to the formation of human capital. When the work of the university refers to the training of scientists and the generation of knowledge in biomedical sciences, almost all the main efforts are funded by public sources, whether state or federal.

To provide greater access and broaden the integration of science in countries of large territorial extent, it is first necessary to understand how they are organised, how they work, and what role they play in teaching and research. The State Public Institutions of Higher Education (SIHE) are those created and maintained by the federation states and are subordinate to the State Education System of their states, in which each SIHE has specific local particularities. The majority of the SIHE have no tax revenue to compose their budgets. These institutions, which must simultaneously develop teaching, research and outreach programs, suffer from inequality and differential access to resources due to budget cuts and re-prioritisation of funding.^14^ ^,^ ^15^

In Brazil there are 129 SIHE in operation, 39 of which are Brazilian State Universities (SUs), such as multidisciplinary academic institutions that have institutionalised intellectual production. Moreover, they can define functions such as presenting minimum requirements for academic degrees, faculty workload, autonomy to create courses, academic and administrative headquarters, issue diplomas, set curricula and number of vacancies, sign contracts, agreements, and covenants, among other actions, respecting the current laws and the constitutional norm.^16^ ^,^ ^17^ ^,^ ^18^

The SUs are distributed among 23 Brazilian states and only nine states allocate state resources to the SIHE linked to taxes, above what is defined by the Federal Constitution of 1988. But since 2013 the resources allocated to the SUs have been decreasing, and consequently leading to an increase in the precariousness of the SUs.^16^ ^,^ ^17^ In this perspective, SIHE must face different challenges due to current globalisation trends. In fact, universities are dealing each year with different measurements as quantification of their progress in different parts worldwide. For this reason, a list of the top universities is always released, which quantifies different aspects that must be considered. For example, the formation of advanced human capital, research, development/innovation, and international collaborations, reveal the competitive advantage for the nation and its organisations.

Regarding the technological and scientific development of the 70 Brazilian SIHE that make up the Times Higher Education World University Rankings 2022, the Universidade de São Paulo (range 201-250) and the Universidade Estadual de Campinas (range 401-500) rank first and second, respectively.^19^ It is important to note that São Paulo is the state with the highest investments in SUs, followed by Paraná, Rio de Janeiro, and Bahia. It is also noteworthy that many of the resources for such development come mainly from research grants funded by national public sources and less often by private and international ones.^14^ ^,^ ^15^ ^,^ ^17^ Thus, even not being among the highest ranked in the world, the SUs mark their presence with relevant technological and scientific contributions, in addition to their fundamental importance for local development. Despite the difficulties faced by the SIHE, according to the 2020 higher education census, the state network recorded growth in enrollment of 3.8% in the period from 2010 to 2020.^18^

In other Latin American countries, the situation is not very different. We could cite several countries, but we will only give the example of Chile because it is representative of the regional situation. In Chile, there are 61 universities, of which 30 belong to the rector’s board.^20^ Within them, there are three categories. Public universities, which are partially financed with government funds; long-established private universities, and more recently, formed private universities.^21^ However, biomedical research and access to funds for this field are mainly concentrated in universities in the central-southern region of the country. There are no private entities that finance biomedical research and only governmental funds from the National Research and Development Agency (ANID) finance a small number of research grants.^22^

The home lesson of the pandemic leads us to insist on a national policy of incentive for education and science. What situation is currently in Brazil? Current financial support situation

Despite several difficulties, Brazil is a country that has a structured Education and Science system that depends on the continuous talks of stakeholders within the ecosystem and on a state policy that withstands the democratic change of governments. There is no reason to reinvent the wheel every four years. The state must be the inducer of the education of the population from an early age, through the valorisation of teachers (salaries, continuing education, career plan) as well as the structuring of basic schools (with laboratories, libraries, internet access, school meals, and parent participation) to higher education (investment in scholarships, teaching careers, and structuring). For this, it has the Ministries of Education and Science and Technology and their autarchies (CAPES, CNPq, FINEP).

Budgets for education and science should not be considered expenditures, but an investment. The short-sighted culture of several governments and some sectors of society, which suffer from the desire for immediacy and the lack of scientific knowledge, fail to see that investing in science and education is not an expense. These investments can lead to a more prosperous future for the nation since they can create an entire productive and innovative ecosystem that reduces Brazil’s dependence on external technologies. Due to the lack of state policy and the continuous decrease and contingency of available resources for Science, Brazil is investing less in the actors, leading to brain drain and the scrapping of the structure formed over more than 50 years of research and graduate studies in Brazil.

The situation in other Latin American countries is not much better. According to data from the World Bank^23^ the average investment in science and technology in Latin America and the Caribbean averages 0.67% of the gross domestic product. Even in these circumstances, Brazil has a leading position, investing more than any other economy in the region (1.21), followed far behind by Cuba (0.55), Argentina (0.46), and Ecuador (0.44). In other countries, R&D investment is relatively low as in Costa Rica (0.37), Chile (0.34), Uruguay (0.32), and Colombia (0.32).^23^

However, as seems obvious, increasing R&D spending is a political decision that is closely related to the economic situation of each country and to the vision of the ruling class to have the political will to do so. However, this may not be enough. It is also necessary for society to value scientific activity and to perceive it as something transcendent for the country. The society should understand that if is necessary to invest in the construction of bridges, roads, and social housing, it is also imperative to invest in science and technology. To achieve this, it is advisable that scientists establish channels of communication and dissemination with society, so that society first learns about the impact of science on society and then understands and values scientific activity. In this respect, it seems that society was able to perceive the value of science better than ever during the COVID-19 pandemic. However, it is important to keep up and reinforce the message, especially in our countries where decisions are often driven by immediacy and social and political contingency.

The lack of investment affects the main researcher (intellectual and financial manager of the research line) as well as those who can be considered the workers of science in Brazil, and graduate students. There was a time when students could participate in courses and conferences in their areas of knowledge in person. This, however, had to be changed both by the circumstances of the COVID-19 pandemic that we are experiencing and by the lack of support for participation in events, increasingly expensive (due to cost increases), organised by the scientific society and higher education institutions. In this way, it is urgent to return support for students to levels of previous decades and the investment of Scientific Society and IES in events that if not only in a virtual way, at least hybrid, are able to reach researchers and students from all corners of the world.

Learning during the pandemic. Using a remote system to integrate students and researchers

With the appearance of the pandemic, a scenario of uncertainty grew with the lockdown and the frightening epidemiological situation of infected and dead throughout the world. In this situation of inequality, where investment in science depends on compliance with the laws of each state, the initiatives of seminars, workshops, and courses remotely during the pandemic allowed students and researchers from different places and with different levels of training to meet each other, share knowledge and scientific discussions and start collaboration without financial or geographical limitations.

How much impact on the indices of scientific growth could have initiatives of remote activities integrating groups far from large centers? In particular, how much growth and quality in science will there be in the area of host-pathogen interaction with an effort to expand for a multidisciplinary vision? Could the pandemic challenge researchers to find new ways to teach and learn in the area of host-pathogen interaction? Towards an integrated education with active methods

The COVID-19 pandemic challenged us not only in healthcare, the most affected initially, but also in the economic, social, cultural, and educational spheres. Educators had to devise, create, or use strategies to gain and keep their students’ attention. Student engagement by applying active learning tools leads to effective gain in which the students are a central piece of all learning processes and drive one another to actively construct their knowledge.^24^ Additionally, using different active learning tools it is possible to reach diverse degrees of student comprehension as observed by McGreevy and Church.^25^ In this way, internationalisation actions and the use of information and communication technologies (ICTs) have the potential to supplement, enrich and improve education.^26^

In times of pandemic crisis, it has been very interesting to review the methods of teaching. Passive methods, hardly interactive and based on transmission of knowledge through books, have given way to active methods. In current methods the students/researchers assume a leading role, allowing creativity to stimulate the search for knowledge and novel strategies to solve scientific problems.

Training of creative human resources, with technical, ethical and communication skills, as well as research and group-based capacity should be a priority to achieve a globalised and critical professional who is able to interact with scientific partners around the world.

We have used the strategy of active methods in seminars, courses, and workshops in the area of host-pathogen interaction, taking advantage of the multi-disciplinary nature of the field.

Critical thinking is imperative in a society that needs to deal with the diversity^27^ present in the 21st Century. This requires adaptiveness, complex communication, social competencies, non-routine problem solving, self-management, personal development, and systemic thinking.^28^

The concept of active citizenship is an emergent concern mainly in developing countries. According to White,^29^ a competent active citizen implies participation in the public sphere. Finally, successful, credible and committed performance in the context of Science, Technology and Innovation depends on continuous updates to knowledge and the generation of impactful scientific research, which necessitates the formulation of efficient public policies that favor the solution of regional and even national problems.

In conclusion

Host-Pathogen interaction is a concept that allows the integration of multiple disciplines improving teaching, learning and research in biological sciences. The scientific community, in times of a pandemic, creates seminars and workshops with the remote transmission, which made it possible to bring research groups together, reduce regional isolation and expand the country’s collaboration network. In times of economic crisis, the use of a platform, at low cost, that allows greater scientific integration, without the need for physical presence, benefits students and researchers. A politic of higher access and integration from different fields of biomedical sciences will increase quantity, quality and perspectives to get top level in sciences.

## References

[1] Ramírez MI, Ruiz RC, Santos AGA, Ungri AM, Almeida BR, Sabatke B Host-Pathogen Interaction Meeting (HPIM) 2021: a virtual event organized by the Brazilian scientific community. Wiley online Library.

[2] Morens DM, Fauci AS (2020). Emerging pandemic diseases how we got to COVID-19. Cell.

[3] Bhutta ZA, Sommerfeld J, Lassi ZS, Salam RA, Das JK (2014). Global burden, distribution, and interventions for infectious diseases of poverty. Infect Dis Poverty.

[4] Booth M (2018). Climate change and the neglected tropical diseases. Adv Parasitol.

[5] WHO (2015). Investing to overcome the global impact of neglected tropical diseases. http://www.who.int/neglected_diseases/9789241564861/en/.

[6] Pecoul B, Chirac P, Trouiller P, Pinel J (1999). Access to essential drugs in poor countries a lost battle?. JAMA.

[7] Moran M (2005). A breakthrough in R&D for neglected diseases new ways to get the drugs we need. PLoS Med.

[8] Casadevall A, Fang FC (2020). The intracellular pathogen concept. Mol Microbiol.

[9] Osorio L, Ríos I, Gutiérrez B, González J (2012). Virulence factors of Trypanosoma cruzi who is who?. Microbes Infect.

[10] Singer M (2010). Pathogen-pathogen interaction a syndemic model of complex biosocial processes in disease. Virulence.

[11] Zamith-Miranda D, Nimrichter L, Rodrigues ML, Nosanchuk JD (2018). Fungal extracellular vesicles modulating host-pathogen interactions by both the fungus and the host. Microbes Infect.

[12] Méthot PO, Alizon S (2014). What is a pathogen Toward a process view of host-parasite interactions. Virulence.

[13] Karlsson EK, Kwiatkowski DP, Sabeti PC (2014). Natural selection and infectious disease in human populations. Nat Rev Genet.

[14] Casadevall A, Pirofski LJ (2001). Host-pathogen interactions the attributes of virulence. Infect Dis.

[15] Carvalho RRS, Amaral NC (2020). Universidades estaduais brasileiras diversidade acadêmica, classificações institucionais e normativa. Rev Práxis Educ.

[16] Conceição FCM, Novais VSM, Castro AS (2020). The financing of Brazilian state universities a review of academic production from 2000 to 2018. Braz J Develop.

[17] BRASIL (1988). Constituição da República Federativa do Brasil: 1988. Diário Oficial da União, Brasília,.

[18] Carvalho RRS, Amaral NC (2020). Universidades estaduais brasileiras financiamento, desigualdades regionais e o PNE (2014-2024). Interação.

[19] Inep - Instituto Nacional de Estudos e Pesquisas Educacionais Anísio Teixeira (2022). Sinopse estatística da educação superior 2020.

[20] THE (2022). World University Rankings. https://www.timeshighereducation.com/world-university-rankings/2022/worldranking#!/page/0/length/25/locations/BRA/sort_by/rank/sort_order/asc/cols/stats.

[21] CRUCH (2023). Chile: Consejo de Rectores Online. https://www.consejoderectores.cl/el-consejo/rectores-del-cruch/.

[22] Ayuda MINEDUC Atención Ciudadana (2023). Portal de Atención Ciudadana del Ministerio de Educación del Gobierno de Chile [homepage on the Internet]. Mecanismos de financiamiento de la educación superior.

[23] Boisier ME, Cevallos RA (2019). Instrumentos de fomento para la investigación en Chile historia reciente, estado actual y desafíos. Rev Med Clin Condes.

[24] World Bank (2019). Gasto en investigación y desarrollo (% del PIB) - Latin America & Caribbean. https://datos.bancomundial.org/indicator/GB.XPD.RSDV.GD.ZS?locations=ZJ.

[25] Carr R, Palmer S, Hagel P (2015). Active learning the importance of developing a comprehensive measure. Act Learn High Educ.

[26] McGreevy KM, Church FC (2020). Active learning subtypes, intra exam comparison, and student survey in an undergraduate biology course. Educ Sci.

[27] Nussbaum MC (2006). Education and democratic citizenship capabilities and quality education. J Hum Dev.

[28] National Research Council Exploring the intersection of science education and 21st century skills: a workshop summary. The National Academies Press.

[29] White M (2013). Higher education and problems of citizenship formation. J Philos Educ.

